# Docosahexaenoic Acid Reverted the All-*trans* Retinoic Acid-Induced Cellular Proliferation of T24 Bladder Cancer Cell Line

**DOI:** 10.3390/jcm9082494

**Published:** 2020-08-03

**Authors:** Lara Costantini, Romina Molinari, Barbara Farinon, Veronica Lelli, Anna Maria Timperio, Nicolò Merendino

**Affiliations:** Department of Ecological and Biological Sciences (DEB), Tuscia University, Largo dell’Università snc, 01100 Viterbo, Italy; rominamolinari@libero.it (R.M.); barbara.farinon@gmail.com (B.F.); v.lelli@unitus.it (V.L.); timperio@unitus.it (A.M.T.)

**Keywords:** all-*trans* retinoic acid, cellular proliferation, T24 bladder cancer cell line, docosahexaenoic acid, palmitic acid, linoleic acid

## Abstract

The treatment of solid cancers with pharmacological all-*trans* retinoic acid (ATRA) concentrations, even if it is a gold standard therapy for the acute promyelocytic leukaemia (APL), is not always effective due to some resistance mechanisms. Here the resistance to ATRA treatment of T24 cell line, bladder cancer, was investigated. T24 was not only resistant to cell death when treated at concentrations up to 20 µM of ATRA, but it was also able to stimulate the cellular proliferation. An over-expression of the fatty acid binding protein 5 (FABP5) in conjunction with the cellular retinol-binding protein-II (CRABP-II) down-expression was found. However, the direct inhibition of the peroxisome proliferator-activated receptor β/δ (PPARβ/δ) did not abolish T24 proliferation, but rather potentiated it. Moreover, considering the ability of the long-chain fatty acids (LCFAs) to displace ATRA from FABP5, the actions of the saturated palmitic acid (PA), unsaturated omega-6 linoleic acid (LA) and omega-3 docosahexaenoic acid (DHA) were evaluated to counteract ATRA-related proliferation. ATRA-PA co-treatment induces cellular growth inhibition, while ATRA-LA co-treatment induces cellular growth enhancement. However, even if DHA is unsaturated LCFA as LA, it was able to reverse the ATRA-induced cellular proliferation of T24, bringing the viability percentages at the levels of the control.

## 1. Introduction

Urinary bladder cancer causes approximately 550,000 new cases annually in the world, that are the 3.0% of all new cancer diagnoses, and 2.1% of all cancer deaths [1]. Among the geographical area, Europe and North America record a three-fold higher rates of incidence, probably due to the risk factors’ exposure as cigarette smoking, chemical carcinogens, arsenic drinking water, obesity, and chronic urinary infections caused by *Schistosoma hematobium* [2]. Among all bladder cancer cases, transitional cell carcinoma (TCC) accounts for 95% of them. TCC is a malignant cancer that originates from the bladder transitional epithelial cells, and often establishes the metastatic disease. It should be note that although from 2000 the TCC incidence-based mortality has started to decrease, these data exclude the mortality cases due to TCC that causes distant metastasis, which has continued to increase since 2012 [3].

ATRA is one of the most biologically-active forms of vitamin A in cells. It influences cellular growth and differentiation in several ways, but mostly by transcriptionally regulating gene expression by binding to nuclear retinoic acid receptors (RARs) and retinoid X receptors (RXRs) [4]. From the U.S. Food and Drug Administration approval in 1995, the ATRA oral administration, at pharmacological doses (45 mg/m^2^/day for adults, in comparison to 900 and 700 μg/day of average requirement for adult men and women, respectively), has revolutionized the treatment of APL, increasing the complete remission rate up to 90–95% [5]. Despite the high complete remission rates, the relative contribution of maintenance therapy (i.e., mostly 45 mg/m^2^/day oral ATRA treatment for 15 days every 90 days for 2 years) is controversial, mostly for high-risk patients (i.e., white cell count > 10 × 10^9^/L) [6]. However, beyond the close connection with APL, pharmacological ATRA treatment may have different systemic effects. Indeed, it is well known that in the treatment of solid cancers the ATRA action is questionable. Although, ATRA can inhibit cancer cell proliferation at different levels, from membrane to gene expression, in parallel the cancer cells have evolved several escape routes to bypass these ATRA actions, developing ATRA-resistance [4]. In some cases, molecular pathways deregulations reverse the ATRA action from the growth inhibitory/differentiating to the anti-apoptotic/proliferative action. Indeed, for example in the classical pathway, ATRA in the cytosol is tied to CRABP-II, which delivers it to RARs. However, it was shown that some solid cancers activate a non-genomic pathway due to less CRABP-II concentration in comparison to another retinol-binding protein, FABP5. In these cases, FABP5 delivered ATRA to the nuclear PPARβ/δ stimulating cellular proliferation [4,7]. Although few papers showed that the maintenance treatment of APL with ATRA is not associated with higher incidence of secondary cancers, it is worth noting that long-term follow-up studies that analyze the APL maintenance therapy adverse effects are actually limited and/or examined a very limited statistical population [8,9].

T24 is a TCC cell line, grade 3, already studied in the literature in correlation to pharmacological ATRA treatment, even if with conflicting results. Indeed, already in 1999 Waliszewski and co-workers failed to found viability inhibition in T24 cell line after 10 µM ATRA and 13-*cis* retinoic acid (13-*cis* RA) treatments [10]. In the same paper the authors found undetectable or low levels of RARβ, cellular retinol-binding protein type-I (CRBP-I), CRABP-I, and CRABP-II in the constitutive and stimulated (48 h 10 µM ATRA stimulation) T24 cells [10]. Zou and co-workers in the paper of 2001, found the same result [11]. Conversely, in the following paper by Zou and co-workers, a different result was found: Indeed increasing the ATRA concentration (from 1 to 10 µM) increased the growth inhibitory effect in T24 cell line, even if the apoptosis induction was similar to control [12].

Considering the discrepancies present in the literature, the present paper evaluated the hypothesized ATRA resistance of T24 cell line, finding that ATRA activates survival mechanism inducing T24 cell proliferation. First investigations about molecular mechanism were carried out analyzing the FABP5-PPARβ/δ pathway, and even if over-expression of FABP5 was found concomitantly with CRABPII down-expression, the direct inhibition of PPARβ/δ did not abolish T24 cellular proliferation, but rather potentiated it. Moreover, considering the paper by Levi [13] and colleagues that demonstrated the action of LCFAs to displacing ATRA from FABP5, where saturated LCFAs inhibit FABP5-PPARβ/δ pathway, while conversely, unsaturated LCFAs activate it, the actions of the saturated PA, unsaturated omega-6 LA, and unsaturated omega-3 DHA were evaluated to counteract ATRA-related proliferation. In line with Levi and co-workers we found that ATRA-PA co-treatment induces cellular growth inhibition and ATRA-LA co-treatment induces cellular growth enhancement [13]. However, conversely to the generalization stated from Levi and co-workers that unsaturated LCFAs induce cell proliferation [13], we found that the unsaturated omega-3 DHA reverse the ATRA-induced cellular proliferation of T24 bladder cancer cell line.

## 2. Materials and Methods

### 2.1. Cells and Treatments

T24 TCC line was purchased from ATCC (Manassas, VA, USA) and cultured in McCoy’s 5A medium supplemented with 10% FBS or FBS charcoal-stripped (FBS-CS), 1.5 mM L-glutamine, and 10 mL/L penicillin/streptomycin, at 37 °C under 5% CO_2_ (at most 5 passages—three days in culture each—from the original ATCC stock). Cells were daily treated with: ATRA 1, 10, and 20 µM; synthetic retinoid fenretinide or 4-HPR (4-hydroxy(phenyl)retinamide) 10 µM; GSK0660 selective PPARδ antagonist 2.5, 5, 10, 20, 30, and 50 µM; PT-S58 (BOC Sciences, Shirley, NY, USA) full PPARβ/δ antagonist 5, 10, 15, and 20 µM; PA, LA, and DHA 10 µM. Controls were done with the corresponding vehicles (DMSO for ATRA, and ethanol for LCFA) in the same percentage present in the treatments. Treatment lapses are reported in the figures for each analysis, anyway with a maximum of 72 h or 96 h. All materials were purchased from Merck (Darmstadt, Germany) except when differently indicated.

### 2.2. Cellular Viability and Proliferation Tests

MTT proliferation assay was used to analyze cellular viability. Briefly, 1500 cells were plated in a 96-well plate in replicates (*n* = 12) and indicated treatment. Cells were incubated with MTT reagent (5 mg/mL) until the formation of formazan crystal (3.5 h). Crystals were dissolved in 1 M HCl in isopropanol, and absorbance at 570 nm was measured using a microplate reader (Infinite 2000, Tecan, Männedorf, Switzerland).

Colony formation assay was used to analyze cellular proliferation. A bottom layer of 0.8% agarose in cell media was cast, in sterile hood, in a six-well plate and set at room temperature to solidify. Cells were suspended in 0.25% agarose in media, 2 mL from this mixture containing 4500 cells was added to each well, and after solidification, 1 mL media with vehicle or treatment was added in each well. Cells were cultured for 21 days. Media were replenished every 3 days. Colonies were visualized by staining with 0.005% crystal violet and counted under a light microscope. The images were acquired through ChemiDoc XRS + (BioRad, Hercules, CA, USA).

Absolute cell count was made through NovoCyte flow cytometer (ACEA Biosciences Inc., San Diego, CA, USA) with Novo Express Software (ACEA Biosciences Inc., San Diego, CA, USA) on T24 cells DMSO- and 10 µM ATRA-treated at 72 h.

### 2.3. Differential Proteomics Analysis

#### 2.3.1. Protein Extraction and Solubilization

DMSO and ATRA treated cells were collected and centrifuged at 1500 rpm for 5 min. Cells were suspended in lysis buffer (7 M urea, 2 M tiourea, 4% *w*/*v* CHAPS, 40 mM Tris-HCl, 0.1 mM EDTA, 1 mM DTT, 50 mM NaF, and 0.25 mMNa_3_VO_4_). The mixture was centrifuged at 12,000× *g* at room temperature for 15 min and a small aliquot was used to determine the protein content by 2D-Quant Kit (GE Healthcare, Chicago, IL, USA).

#### 2.3.2. 2D Gel Electrophoresis and Image Analysis

To remove lipids, 500 μg of protein were precipitated from a desired volume of each sample with a cold mix of tri-n-butyl phosphate/acetone/methanol (1:12:1). After incubation at 4 °C for 90 min, the precipitate was pelleted by centrifugation at 2800 ×*g*, for 20 min at 4 °C. The pellet was air-dried and then solubilized in the buffer containing 7 M urea, 2 M thiourea, 4% (*w*/*v*) CHAPS, 40 mM Tris-HCl. The sample was subsequently reduced (5 mM tributylphosphine, 1 h) and alkylated (7.7 Mm IAA, 1 h). To prevent over-alkylation, iodoacetamide (IAA) excess was neutralized by adding 10 mM DTE. Sample is included in the rehydration solution (7 M Urea, 2 M Thiourea, 4% CHAPS and 0.5% *w*/*v* pH 4–7 carrier ampholyte (Bio-Rad, Hercules, CA, USA)) and the sample is taken up into the 17 cm IPG strips pH 3–10 (Bio-Rad, Hercules, CA, USA) passively during rehydration overnight. IEF was performed using Bio-Rad Multiphore II and Dry Strip Kit (Bio-Rad-Protean IEF-Cell-System). Seventeen centimeters IPG strips (Bio-Rad, Hercules, CA, USA) pH 4–7 were passively rehydrated overnight with 500 μg of protein in 300 μL of solubilization solution (7 M Urea, 2 M Thiourea, 4% CHAPS and 0.5% *w*/*v* pH 4–7 carrier ampholyte (Bio-Rad, Hercules, CA, USA)). The total product time × voltage applied was 80,000 V h for each strip at 20 °C. Strips were equilibrated (30 min in 50 mM Tris-HCl pH 8.8, 6 M urea, 30% glycerol *v*/*v*, 1% SDS, bromophenol blue) and then placed on SDS-polyacrylamide gels, 18.5 cm × 20 cm, 13% acrylamide, and sealed with 0.5% agarose. SDS-PAGE was performed using the Bio-Rad Protean II XL Cell, large gel format, at constant current (40 mA per gel) at 7 °C until the bromophenol blue tracking dye was approximately 2–3 mm from the bottom of the gel. Protein spots were stained with colloidal Coomassie Brilliant Blue G-250. To ensure protein pattern reproducibility, two technical replicates for each biological replicate were done. The scanned gel images were transferred to the Progenesis SameSpots software package (Nonlinear Dynamics, Newcastle, UK), which allows spot detection, background subtraction, and protein spot OD intensity quantification (spot quantity definition). The gel image showing the highest number of spots and the best protein pattern was chosen as a reference template and the images were aligned onto it. All spots were prefiltered and manually checked before applying the statistical criteria (ANOVA *p* < 0.05 and fold ≥ 1.5).

#### 2.3.3. In-Gel Digestion and LC-MS/MS Analysis

Gel bands were carefully excised from the gel and subjected to in-gel trypsin digestion according to Shevchenko and colleagues [14]. Peptide extracts were analyzed by using a split-free nano-flow liquid chromatography system (EASY-nLC II, Proxeon, Odense, Denmark) coupled with a 3D-ion trap (model AmaZon ETD, Bruker Daltonik, Billerica, MA, USA) equipped with an online ESI nanosprayer (the spray capillary was a fused silica capillary, 0.090 mm OD, 0.020 mm ID) in the positive-ion mode. For all experiments, a sample volume of 15 μL was loaded by the autosampler onto a homemade 2-cm fused silica precolumn (100 μm I.D.; 375 μm O.D.; Reprosil C18-AQ, 5 μm, Dr. Maisch GmbH, Ammerbuch-Entringen, Germany). Sequential elution of peptides was accomplished by using a flow rate of 300 nL/min and a linear gradient from Solution A (100% water; 0.1% formic acid) to 50% of Solution B (100% acetonitrile; 0.1% formic acid) in 40 min over the precolumn on line with a homemade 15 cm resolving column (75 μm ID; 375 μm OD; Reprosil C18-AQ, 3 μm, Dr. Maisch GmbH, Ammerbuch-Entringen, Germany). The acquisition parameters for the mass spectrometer were as previously reported. Acquired MS/MS spectra were processed in DataAnalysis 4.0 and submitted to the Mascot search program (Matrix Science, London, UK). The following parameters were adopted for database searches: SwissProt database (release date 7 April 2017; 5,011,440 sequences); taxonomy = Homo sapiens; peptide and fragment mass tolerance = ±0.3 Da; missed cleavages = 1; fixed modifications = carbamidomethyl (C); variable modifications: oxidation (M) and significance threshold level (*p* < 0.05) for Mascot scores (−10 Log (P)). In the case of hits with only one statistically significant unique peptide, even though high Mascot scores were obtained with significant values, a combination of automated database searches and manual interpretation of peptide fragmentation spectra was used to validate protein assignments.

For the analysis of functional relationships between the proteins that exhibited differences in expression String DB was used.

### 2.4. Quantitative Real-Time PCR

T24 RNA was extracted from 10^6^ cells using the “high pure RNA isolation kit” (Roche, Basel, Switzerland) and quantified through Qubit 3.0 Fluorometer (Life Technologies, Carlsbad, CA, USA) and Qubit RNA BR Assay kit (Life Technologies, Carlsbad, CA, USA). cDNA was generated using the “transcriptor universal cDNA master” (Roche, Basel, Switzerland). Quantitative real-time PCR was carried out using the “LightCycler 480 SYBR Green I Master” (Roche, Basel, Switzerland) on LightCycler 480 instrument (Roche, Basel, Switzerland), with the following primer pairs (TIB MOLBIOL S.r.l., Genova, Italy):

CRABP-II:

Forward primer—ATGCCCAACTTCTCTGGCAA

Reverse primer—CGTCATGGTCAGGATCAGTT

FABP5:

Forward primer—AGCAGCTGGAAGGAAGATGG

Reverse primer—CTGATGCTGAACCAATGCAC

β-actin:

Forward primer—GCATGGAGTCCTGTGGCAT

Reverse primer—CTAGAAGCATTTGCGGTGG

For each treatment three technical replicates of each three biological replicates were performed (*n* = 9). As calibrator RNA of non-cancerous human urothelial cells (HUC) was used (ScienCell Research Laboratories, Carlsband, CA, USA). Relative expression was calculated by the E-Method, with β-actin as the reference.

### 2.5. Immunoblotting

Total cell proteins were extracted from T24 cell line using a buffer containing 250 mM TRIS-HCl pH 7.5, 750 mM NaCl, 25 mM EDTA, 10% glycerol, 1% IGEPAL CA-630, and 1% protease inhibitors. Non-cancerous HUC lysate was purchased from ScienCell Research Laboratories (Carlsband, CA, USA). Human bladder normal tissue lysate total protein was purchased from Abcam (Cambridge, UK). The protein concentration of the supernatants was determined with the BCA protein assay. 40 µg of proteins were resolved by electrophoresis on SDS-PAGE precast gels (4–20% mini protean TGX gels, BioRad, Hercules, CA, USA) and transferred onto nitrocellulose membrane (Amersham, GE healthcare, Chicago, IL, USA). Membranes were incubated with primary antibodies, followed by washes with Tween-PBS, and incubation with horse-radishperoxidase-conjugated antibodies. Protein expression was detected by exposure to westar supernova (Cyanagen, Bologna, Italy) and the images were collected through ChemiDoc XRS + (BioRad, Hercules, CA, USA). Antibody against CRABP-II II was purchased from Thermo Fischer scientific (Waltham, IL, USA), antibodies against, FABP5 and β-actin were purchased from Santa Cruz Biotechnology (Dallas, TX, USA), and they were used at 1:500 dilutions for CRABP-II and FABP5, and 1:1000 dilution for β-actin. Densitometry of various analyzed proteins and their respective loading controls from the same blot was performed using ImageJ Software (https://imagej.nih.gov/ij/index.html).

### 2.6. Flow-Cytometer Analysis of Apoptosis

Apoptosis was determined with annexin V-FITC/propidium iodide (PI) apoptosis detection kit (Bender MedSystems, Wien, Austria). Following treatment, aliquots containing 5 × 10^5^ cells in 195 µL buffer were stained with 5 µL FITC-conjugated Annexin V for 10 min and after washing and resuspension in 190 µL buffer, cells were stained with 10 µL PI solution. Samples were stored on ice until data acquisition. The analysis was performed by NovoCyte flow cytometer (ACEA Biosciences Inc., San Diego, CA, USA) with Novo Express Software (ACEA Biosciences Inc., San Diego, CA, USA).

### 2.7. Statistical Analysis

The mean and standard deviation (SD) of at least three biological replicates were calculated for all the analyzed data. Statistical analysis was performed with the XLSTAT 2018.5 (Addinsoft SARL, Paris, France) software using one-way ANOVA. Fisher’s least significant differences test was used to describe statistical differences between means at the *p* < 0.05 significance level.

## 3. Results

### 3.1. ATRA Induce Cellular Proliferation of T24 Cell Line

**Figure 1:**
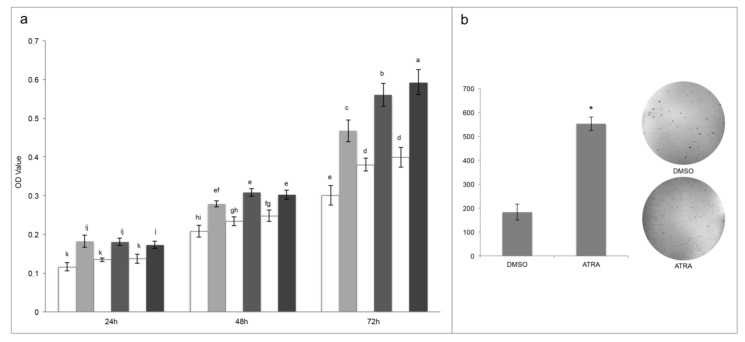
All-trans retinoic acid (ATRA) treatments induce T24 proliferation. (**a**) MTT assays express as optical density (OD) values in T24 cells treated with different consecutive concentration of ATRA (grey scale: 1 μM, 10 μM, and 20 μM) or respective concentration of vehicle DMSO (white: 0.002%, 0.02%, and 0.4%) (for 3 days). Means ± SD (*n* = 12). Means with different letters are significantly different (*p* < 0.05). (**b**) Colony formation assay. Representative image (out of 3) of colonies formed in soft agar by T24 cells treated with vehicle (DMSO) or ATRA (10 μM). Means ± SD (*n* = 3), * indicates the statistically significant difference.

As reported above, discordances are present in the literature in relation to the action of ATRA treatment in T24 cells so, first of all, the resistance of T24 cell line after ATRA treatment was evaluated. As reported in the Figure 1, panel a, T24 cells are significantly resistant to the cytotoxic effect of ATRA at the different treatment times (24 h, 48 h, and 72 h) and at all concentrations used (1, 10, 20 μM) tested with MTT viability assay (Figure 1a). Moreover, daily treatment did not cause toxicity for ATRA accumulation in the cells, but rather it stimulated a greater significantly metabolic activity, which therefore was time and dose dependent (from 24 h to 72 h), in comparison to the vehicle DMSO in the same concentrations of the treatments (0.002%, 0.02%, and 0.4% for ATRA 1 μM, 10 μM, and 20 μM, respectively) (Figure 1a). Indeed, the higher values were found at 72 h of treatments with a range of viability between 47–55% for the three treatment doses in comparison to their respective controls (Figure 1a). These proliferation rates were also confirmed by an absolute cell count made through flow-cytometry, which identified a cell number 52% higher in 10µM ATRA-treated T24 at 72 h in comparison to the vehicle DMSO-treated cells (Supplementary Figure S1). Even if the three different rising ATRA concentrations were analyzed from nutraceutical to pharmacological ones, up to the toxic one, the 10 µM concentration was used in the subsequent analyses to perform comparison with the existing literature on bladder cancer, and to maintain a range similar to APL pharmacological treatment [15].

Despite the rising optical density identified by MTT assay between treatment lapses denoted cellular proliferation, to confirm that ATRA induce proliferation, a colonies formation assay was performed. As reported in the Figure 1, panel b, 10 µM ATRA significantly induced cellular proliferation generating an almost triple number of colonies in comparison to DMSO (553 ± 28 for ATRA in comparison to 183 ± 33 for DMSO; Figure 1b).

**Figure 2:**
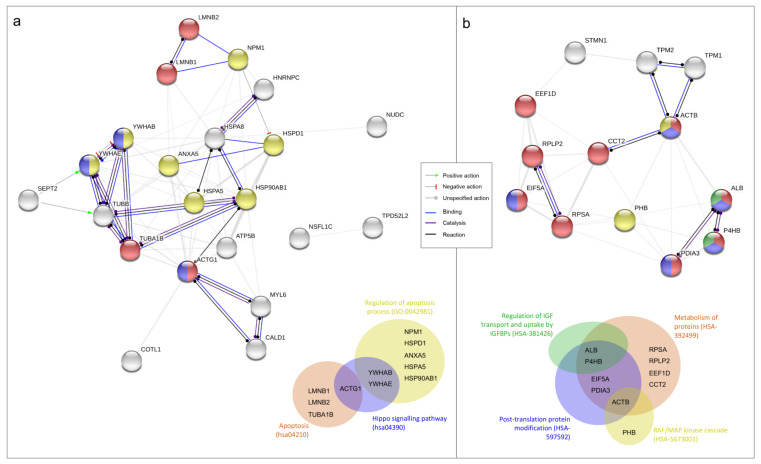
Functional proteins association networks identified by differential proteomic analysis and relative Venn diagrams. Interaction networks identified between under-expressed (panel **a**) and overexpressed (panel **b**) proteins in T24 cells after 3 days (72 h) of 10 µM ATRA treatment in comparison to vehicle treatment (DMSO). All the proteins identified by the proteomic analysis are reported; only the actions (lines between nodes) of the selected pathways are highlighted with the colors reported in the figure’s legend. Different colors of nodes indicate the following different functional relationships, according to STRING database and referring to Gene Ontology (GO), KEGG Pathway (hsa), and Reactome Pathways (HSA); identifier number is reported in the brackets. Nodes of multiple colors indicate belonging to multiple pathways. Venn diagrams summarize the affiliation of proteins to different molecular pathways. (**a**) Yellow nodes: regulation of apoptosis process (GO:0042981); red nodes: apoptosis (hsa04210); blue nodes: hippo signaling pathway (hsa04390). (**b**) Red nodes: metabolism of proteins (HSA-392499); blue nodes: post-translation protein modification (HSA-597592); green nodes: regulation of insulin-like growth factor (IGF) transport and uptake by insulin-like growth factor binding proteins (IGFBPs) (HSA-381426); yellow nodes: RAF/MAP kinase cascade (HSA-5673001).

As further confirmation of the pharmacological ATRA treatment proliferative action, a differential proteomic analysis between DMSO and 10 µM ATRA treated T24 cells was done after 72 h of treatments (treatment time with the found higher proliferative rate). The comparative analysis revealed a total of 87 differentially abundant protein spots (*p* < 0.05; fold change ≥ 1.5; Supplementary Figure S2). Variable spots were excised from the gels, digested by trypsin, and peptide mixtures were then analyzed by LC-ESI-MS/MS for protein identification. The positively identified proteins are listed in the Supplementary Table S1, together with the protein spot number and the identification parameters. The total positively identified proteins were 38, of which 25 down regulated and 13 up regulated (Supplementary Table S1). In order to identify the relevant pathways that cause cellular proliferation after 72 h of daily 10 µM ATRA treatment, the two different protein lists (i.e., down regulated and up regulated proteins) were used as input in STRING database. Among the significantly altered biological process from the down regulated proteins there were the regulation of apoptosis process (yellow nodes in Figure 2a; Gene Ontology, GO pathway *n*. 0042981), apoptosis (red nodes in Figure 2a; KEGG Pathway *n*. hsa04210), and the Hippo signaling pathway (blue nodes in Figure 2a; KEGG Pathway *n*. hsa04390). Contrariwise, among the significantly altered biological process from the up regulated proteins there were the metabolism of proteins (red nodes in Figure 2b; Reactome Pathways *n*. HSA-392499), the post-translation protein modification (blue nodes in Figure 2b; Reactome Pathways *n*. HSA-597592), the regulation of insulin-like growth factor (IGF) transport and uptake by insulin-like growth factor binding proteins (IGFBPs) (green nodes in Figure 2b; Reactome Pathways *n*. HSA-381426), and the RAF/MAP kinase cascade (yellow nodes in Figure 2b; Reactome Pathways *n*. HSA-5673001).

### 3.2. FABP5-PPARβ/δ As Hypothetical Molecular Mechanism of ATRA-Induced Proliferation in T24 Cells

**Figure 3:**
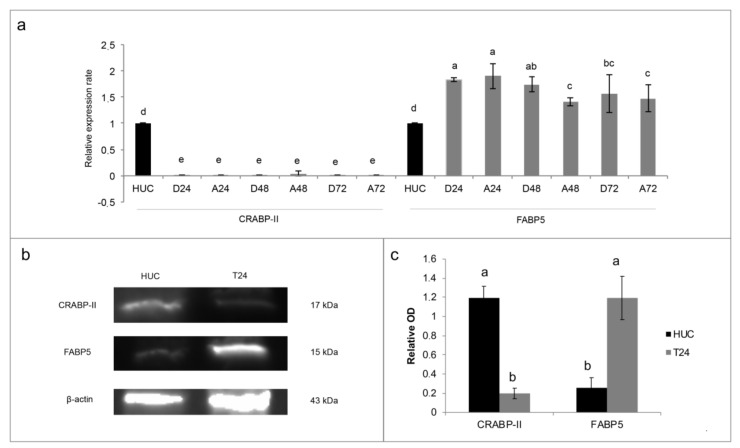
The expression of cellular retinol-binding protein-II (CRABP-II) and fatty acid binding protein 5 (FABP5) in T24 cell line. (**a**) mRNA expression after treatment with 10 µM ATRA for 3 days (72 h). As calibrator mRNA expression of HUC non-cancer cells (human urothelial cells) was used. The expression levels of CRABP-II and FABP5 were normalized to β-actin levels. The data are expressed as means ± SD (*n* = 9). Different letters indicate statistically significant differences. D24, D48, D72: control samples at 24 h, 48 h, and 7 h. A24, A48, and A72: treated samples with 10 µM ATRA for 24 h, 48 h, and 72 h. (**b**) CRABP-II and FABP5 proteins’ expression in untreated T24 and HUC cells. β-actin was the control. Representative image out of three. (**c**) Densitometry analysis of CRABP-II and FABP5 protein expression, normalized to β-actin optical density (OD). The data are expressed as means ± SD (*n* = 3). Different letters indicate statistically significant differences.

**Figure 4:**
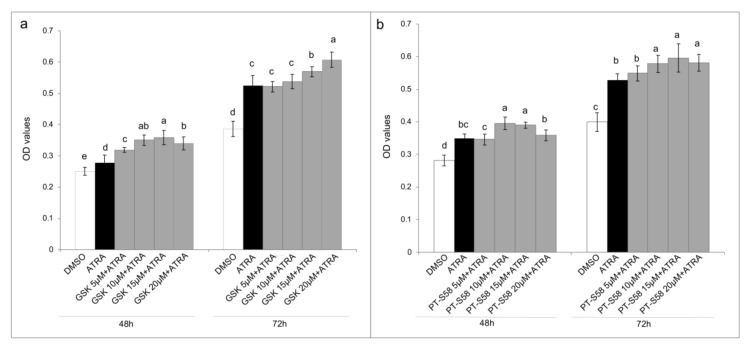
PPARβ/δ inhibition. MTT assays express as optical density (OD) values in T24 cells treated with DMSO, ATRA 10 µM, or ATRA 10 µM added with four GSK0660 (GSK, panel (**a**)) or PTS-58 (panel (**b**)) concentrations (5 µM, 10 µM, 15 µM, and 20 µM), selective and full PPARβ/δ antagonists, respectively, for 3 days (showed 48 h and 72 h results). Means ± SD (*n* = 12). Means with different letters are significantly different (*p* < 0.05) within each treatment lapse.

Even if T24 cell line was resistant to rising concentrations of ATRA treatment, the same resistance does not occur after the treatment with the synthetic retinoid 4-HRP (Supplementary Figure S3). Considering that 4-HRP has an independent cytotoxic mechanism from the classical retinoid receptor pathways, modifications in these pathways could be the cause of the ATRA resistance. For this aims we analyzed both the amount of the expression of CRABP-II (as showed in a previously paper [10]) that the FABP5-PPARβ/δ pathway. As showed in the Figure 3a, CRABP-II mRNA expression was down regulated in comparison to the expression in HUC non-cancer cells. Contrariwise, FABP5 mRNA expression was almost double up regulated in T24 cells (Figure 3a). Even if the 10 µM ATRA treatment for 72 h slightly modified the FABP5 mRNA expression, the same does not happen for the mRNA of CRABP-II, whereby the high FABP5/CRABP-II mRNA ratio was unchanged. The same trend was found for CRABP-II and FABP5 proteins’ expression in T24 cells against HUC (Figure 3b,c), and it was also confirmed against total healthy bladder lysate (Supplementary Figure S3). Likewise of mRNA expression, FABP5 and CRABP-II proteins’ expression were not modulated by 10 µM ATRA treatment for 72 h (Supplementary Figure S4). Considering the validated FABP5-PPARβ/δ pathway and in order to revert ATRA-induced cellular proliferation, PPARβ/δ activity was inhibited with escalating doses (5 µM, 10 µM, 15 µM, and 20 µM) of the two different PPARβ/δ antagonists, GSK0660 and PTS-58, during 10 µM ATRA co-treatment until 72 h (Figure 4 and Supplementary Figure S5). Surprisingly, both the antagonists, dose-dependently and significantly, potentiated the viability increase given by ATRA in the same proportion (12–16% maximum increase at 72 h; Figure 4 and Supplementary Figure S5).

### 3.3. LCFA As Reversion Agents of ATRA-Induced Proliferation in T24 Cells

Considering the paper of Levi [13] and colleagues that demonstrated the action of long-chain fatty acids (LCFA) to displacing ATRA from FABP5, where saturated LCFAs inhibit FABP5-PPARβ/δ pathway, while conversely unsaturated LCFAs activate it, the actions of the saturated PA, unsaturated omega-6 LA, and unsaturated omega-3 DHA were evaluated to counteract ATRA-related proliferation. So, LCFA actions were tested alone in the same concentration of ATRA (10 µM), and with 10 µM of ATRA co-treatment, and all of which in the presence of FBS-CS, deprived of lipophilic molecules.

**Figure 5:**
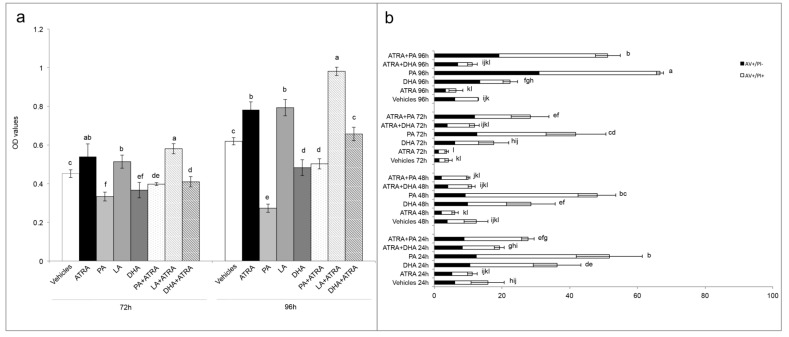
Reversion of ATRA proliferation by long-chain fatty acids (LCFA). (**a**) MTT assays express as optical density (OD) values in T24 cells treated with vehicles (DMSO and ethanol), single treatment of ATRA, PA, LA, and DHA (10 µM) or ATRA 10 µM added with PA, LA, and DHA (10 µM) for 4 days (showed 72 h and 96 h results). Means ± SD (*n* = 12). Means with different letters are significantly different (*p* < 0.05) within each treatment lapse. (**b**) Percentages of total apoptosis (early apoptosis, AV+/PI−; late apoptosis, AV+/PI+) detected by immunofluorescence staining of T24 cells using annexin V-FITC/propidium iodide (PI) staining after single treatment with vehicles (DMSO and ethanol), ATRA, PA, and DHA (10 µM) or ATRA 10 µM added with PA, and DHA (10 µM) for 4 days (96 h). Results are expressed as the mean ± SD of three different experiments. Means with different letters are significantly different (*p* < 0.05).

As expected, T24 cells were PA sensitive, and when co-treatment was present, PA acted as inhibiting agent of ATRA-induced proliferation, with the greatest effect found at 96 h with 36% less viability in comparison to ATRA treatment (Figure 5a). Conversely, LA action was exactly the opposite, leading at 96 h to the same viability rate found with ATRA when LA treatment was administered alone, and giving a 25% potentiation when administered with ATRA co-treatment (Figure 5a). Surprisingly, even if DHA is an unsaturated LCFA as LA, it significantly inhibited T24 viability when administered alone (22% in comparison to vehicles). Therefore, when administered with ATRA co-treatment, DHA inhibited the ATRA-related viability increase bringing the viability rate at similar levels to those of the vehicles (Figure 5a).

Finally, the apoptosis after PA and DHA with ATRA co-treatment was analyzed to understand the LCFA mechanism of ATRA-related proliferation reversion. As showed in Figure 5b, the PA as single treatment or in combination with ATRA, induced the higher percentages of apoptosis with peaks at 96 h of 67% and 51%, respectively. Instead, the action of DHA is different, which while alone produced modest percentages of apoptosis (between 17.5% and 36.5% in 96 h), in combination with ATRA it gave non-statistically different apoptosis rates from those of controls with vehicle during all the 96 h of treatment (Figure 5b).

## 4. Discussion

In the present paper we have shown, for the first time, that T24 cell line are not only resistant to rising ATRA concentrations, up to 20 µM, but also that ATRA induces a dose and time-dependent cellular proliferation (Figure 1b).

As confirmation of ATRA proliferative action in T24 cells a differential proteomic analysis was done comparing cells treated with DMSO and 10 µM ATRA for 72 h. Among the down-regulated proteins reasonably there were proteins belonging to the regulation of apoptosis pathway, but also proteins belonging to the Hippo-signaling pathway. In particular, there was two proteins belonging to the 14-3-3 family (YWHAB and YWHAE), which by binding to YAP and TAZ proteins in the cytosol are able to inactivate the pro-proliferative function of YAP/TAZ inducing cell contact-growth inhibition, necessary to the organ size control [16]. Instead, among the up regulated proteins, beyond those implicated in the metabolism of proteins and in the post-translation protein modifications, obviously necessary to proliferation, others involved in the regulation of IGF transport and uptake by IGFBPs, and in the RAF/MAP kinase cascade were found. IGF exerts a pro-survival action stimulating the PI3k/Akt pathway, and other evidence in the literature showed that in ATRA-sensitive cancer cells, ATRA suppresses IGFs proliferative action, whilst in ATRA-resistant cell lines the IGFs proliferative action occurs for several modified molecular mechanism [4]. In support of this data, previous papers showed the up regulation of the IGFs proliferation pathway in T24 cells in comparison to normal urothelial cells [17,18]. However, here for the first time, it was showed the up-regulation of the IGFs pathway following 10 µM ATRA treatment in T24 cells, which mostly supports the ATRA pro-proliferative action in this cellular model of bladder cancer. Similarly, also proteins of the RAF/MAP kinase pathway turned out to be up-regulated following ATRA treatment in T24 cells. Also in this case, RAF/MAP kinase pathway is inhibited by ATRA treatment in ATRA-sensitive cells, but in ATRA-resistant this pathway is functional and through c-jun and c-fos lead to cellular proliferation [4]. However, the molecular mechanisms by which ATRA up or down regulated the described pathways in T24 cells need to be investigated.

As first step to understand the molecular mechanism behind the ATRA proliferative action in T24 cells, the cytoplasmic retinoid receptors were analyzed, considering that the same resistance found for ATRA did not occur with the synthetic retinoid 4-HRP, which has a cytotoxic mechanism independent from the classical retinoid receptor pathways (Supplementary Figure S2). From the first literature evidence of T24 CRABP-II down-regulation [10], in the present paper the FABP5-PPARβ/δ pathway was for the first time analyzed in the T24 TCC cells and compared with the normal HUC cells. The data found and reported in Figure 3 confirmed our hypothesis: the high FABP5/CRABP-II ratio in T24 cells, in comparison to HUC normal cells, could justify the activation of FABP5-PPARβ/δ non-genomic pathway, which was found to induce proliferation in other ATRA resistant solid cancer cells [4,7]. For this reason, inhibition of PPARβ/δ receptor was attempted through the use of two different antagonists, GSK0660 and PTS-58. However, surprisingly, both the antagonists, dose-dependently and significantly, potentiated the viability increase (Figure 4), even more when ATRA co-treatment was administered, indicating that probably different molecular mechanisms occur. Several assumptions can explain these results from the presence of mutations in the PPARβ/δ receptor, to the interactions with other mutant nuclear molecules that lead to cellular proliferation, until the presence of other nuclear pathways that do not require the PPARβ/δ involvement. In the literature some evidence is present in relation to RXRα [19] and PPARγ [20] genes mutations in bladder cancer that can cause PPARs/RXRα pathway hyperactivation resulting in the cellular proliferation, even though they were not related to ATRA treatments. Moreover, other molecular modifications associated with T24 TCC line and/or bladder cancer tissues have been found and they can be correlated with ATRA resistance as RARβ silencing by aberrant methylation [21], and the absence of the estrogen receptor, or rather estrogen receptor negative (ER-) [22,23]. Finally, even though IGFs and RAF/MAP kinase pathways were here found up-regulated following 10 µM ATRA treatment, further analyses are needed to understand what actually stimulates the cellular proliferation at the nuclear level, especially for retinoids nuclear receptors.

Finally, with the aim to better understand the molecular mechanism of ATRA in relation to FABP5-PPARβ/δ pathway, and in order to find an antagonist that inhibits the ATRA proliferative action, the roles of LCFA was investigated as ATRA co-treatment. Indeed, in the paper of Levi and colleagues [13] was shown that saturated LCFA, in particular PA, is able to displace ATRA from FABP5, inhibiting its delivery to PPARβ/δ, and blocking FABP5 in the cytoplasm. In this way all the ATRA molecules in the cytosol can bind CRABP-II/RARs exerting its classical/genomic activity [13]. Conversely, in the same paper, the authors stated that unsaturated LCFAs, in particular LA, could act similarly to ATRA, binding FABP5, triggering its translocation to the nucleus, and inducing PPARβ/δ activation. However, in the present paper we found that this is an incorrect generalization. Indeed, among the unsaturated LCFAs, DHA was not tested in the paper of Levi and co-workers. In particular, we found similar data for PA and LA, or rather we found that PA reduced viability when administered as co-treatment with ATRA, while LA potentiated the ATRA proliferative action (Figure 5). However, the unsaturated DHA gave different results in comparison to LA, inhibiting the proliferative action of ATRA and giving viability percentages similar to control with vehicles (Figure 5a). The cytotoxic action of DHA was also evident in the DHA single treatment, considering that it induced a significant viability decrease, and significant percentages of apoptosis (Figure 5b). Although a previous paper of Lin and colleagues [23] investigated the combined effect of ATRA and unsaturated LCFA, ALA, EPA, and DHA, on ER- ATRA resistant breast cancer cell lines, they used huge LCFA and ATRA concentrations (80 µM and 20 µM, respectively), and attributed the cytotoxic action to a synergistic action between unsaturated LCFA and ATRA. In the present paper, the reversion of ATRA proliferative action was obtained with the same concentration of DHA and ATRA (10 µM). Moreover, from the data it is possible to speculate that ATRA continues to carry out its proliferative action; however, this is counteracted by DHA co-treatment. Anyway, further investigations are needed to understand the molecular pathways implicated in these effects.

## 5. Conclusions

In the present paper the resistance of T24 cell line to ATRA pharmacological treatment was confirmed and correlated with cellular proliferation. To explain the modified molecular pathway that led to the proliferation, the FABP5-PPARβ/δ pathway was investigated and a high FABP5/CRABP-II ratio was found. However, the inhibition of PPARβ/δ receptor through the antagonists GSK0660 and PTS-58 has not diminished the ATRA cellular proliferation, but even it has enhanced it. Nevertheless, the equimolar DHA and ATRA co-treatment reverted the ATRA-induced T24 cellular proliferation, bringing back to a level comparable to that of the control. In parallel, the highly PA cytotoxic action, and the LA cellular proliferation enhancing-action was confirmed also in T24 TCC bladder cell line, as from previous studies found in the literature done in other cellular models. It is to be noted that contrary to saturated LCFA, the unsaturated DHA has not the same detrimental activities, especially at this low concentration. So, here we speculated that DHA could be potentially safely used as adjuvant in the treatment and maintenance therapy of APL, to counteract the incidence of secondary ATRA-resistant cancers. Further future investigations will be carried out to understand the correct molecular pathways in T24 and in other ATRA-resistant cell lines.

## Supplementary Materials

The following are available online at https://www.mdpi.com/2077-0383/9/8/2494/s1, Figure S1: Absolute cell count made through flow-cytometry of T24 cells DMSO- and 10µM ATRA-treated at 72 h, Figure S2: 2-DE Proteomic profiling of T24 cell line after 10µM ATRA treatment, Figure S3: T24 are resistant to ATRA treatment but not to 4-HRP, synthetic retinoid, Figure S4: CRABP-II and FABP5 proteins’ expression in total healthy bladder lysate, Figure S5: T24 are resistant to PPARβ/δ antagonists, GSK0660 and PTS-58, alone or in combination to ATRA, Table S1: List of differentially abundant proteins identified by LC-MS/MS T24 cells DMSO and 10 µM ATRA treated for three days.

## Abbreviations

13-*cis* RA	13-*cis* retinoic acid
4-HPR	4-hydroxy(phenyl)retinamide
APL	acute promyelocytic leukemia
ATRA	all-*trans* retinoic acid
CRABP-I	cellular retinol-binding protein-I
CRABP-II	cellular retinol-binding protein-II
CRBP-I	cellular retinol-binding protein type-I
DHA	docosahexaenoic acid
ER-	estrogen receptor negative
FABP5	fatty acid binding protein 5
FBS-CS	FBS charcoal-stripped
HUC	human urothelial cells
IGF	insulin-like growth factor
IGFBPs	insulin-like growth factor binding proteins
LA	linoleic acid
LCFAs	long-chain fatty acids
PA	palmitic acid
PI	propidium iodide
PPARβ/δ	peroxisome proliferator-activated receptor β/δ
RARs	retinoic acid receptors
RXRs	retinoid X receptors
SD	standard deviation
TCC	transitional cell carcinoma

## References

[1] GLOBOCAN 2018. http://gco.iarc.fr/.

[2] Richters A., Aben K.K.H., Kiemeney L.A.L.M. (2019). The Global Burden of Urinary Bladder Cancer: An Update. World J. Urol..

[3] Al-Husseini M.J., Kunbaz A., Saad A.M., Santos J.V., Salahia S., Iqbal M., Alahdab F. (2019). Trends in the Incidence and Mortality of Transitional Cell Carcinoma of the Bladder for the Last Four Decades in the USA: A SEER-Based Analysis. BMC Cancer.

[4] Costantini L., Molinari R., Farinon B., Merendino N. (2020). Retinoic Acids in the Treatment of Most Lethal Solid Cancers. J. Clin. Med..

[5] Abaza Y., Kantarjian H., Garcia-Manero G., Estey E., Borthakur G., Jabbour E., Faderl S., O’Brien S., Wierda W., Pierce S. (2017). Long-Term Outcome of Acute Promyelocytic Leukemia Treated with All-Trans-Retinoic Acid, Arsenic Trioxide, and Gemtuzumab. Blood.

[6] McCulloch D., Brown C., Iland H. (2017). Retinoic Acid and Arsenic Trioxide in the Treatment of Acute Promyelocytic Leukemia: Current Perspectives. OncoTargets Ther..

[7] Schug T.T., Berry D.C., Shaw N.S., Travis S.N., Noy N. (2007). Opposing Effects of Retinoic Acid on Cell Growth Result from Alternate Activation of Two Different Nuclear Receptors. Cell.

[8] Muchtar E., Vidal L., Ram R., Gafter-Gvili A., Shpilberg O., Raanani P. (2013). The Role of Maintenance Therapy in Acute Promyelocytic Leukemia in the First Complete Remission. Cochrane Database Syst Rev..

[9] Lou Y., Lu Y., Zhu Z., Ma Y., Suo S., Wang Y., Chen D., Tong H., Qian W., Meng H. (2018). Improved Long-Term Survival in All Sanz Risk Patients of Newly Diagnosed Acute Promyelocytic Leukemia Treated with a Combination of Retinoic Acid and Arsenic Trioxide-Based Front-Line Therapy. Hematol. Oncol..

[10] Waliszewski P., Waliszewska M., Gordon N., Hurst R.E., Benbrook D.M., Dhar A., Hemstreet G.P. (1999). Retinoid Signaling in Immortalized and Carcinoma-Derived Human Uroepithelial Cells. Mol. Cell. Endocrinol..

[11] Zou C., Liebert M., Zou C., Grossman H.B., Lotan R. (2001). Identification of Effective Retinoids for Inhibiting Growth and Inducing Apoptosis in Bladder Cancer Cells. J. Urol..

[12] Zou C. (2006). Comparing the Effect of ATRA, 4-HPR, and CD437 in Bladder Cancer Cells. Front. Biosci..

[13] Levi L., Wang Z., Doud M.K., Hazen S.L., Noy N. (2015). Saturated Fatty Acids Regulate Retinoic Acid Signalling and Suppress Tumorigenesis by Targeting Fatty Acid-Binding Protein 5. Nat. Commun..

[14] Shevchenko A., Wilm M., Vorm O., Mann M. (1996). Mass Spectrometric Sequencing of Proteins from Silver-Stained Polyacrylamide Gels. Anal. Chem..

[15] Muindi J., Frankel S.R., Miller W.H., Jakubowski A., Scheinberg D.A., Young C.W., Dmitrovsky E., Warrell R.P. (1992). Continuous Treatment with All-Trans Retinoic Acid Causes a Progressive Reduction in Plasma Drug Concentrations: Implications for Relapse and Retinoid “Resistance” in Patients with Acute Promyelocytic Leukemia. Blood.

[16] Freeman A.K., Morrison D.K. (2011). 14-3-3 Proteins: Diverse Functions in Cell Proliferation and Cancer Progression. Semin. Cell Dev. Biol..

[17] Sun H.Z., Wu S.F., Tu Z.H. (2001). Blockage of IGF-1R Signaling Sensitizes Urinary Bladder Cancer Cells to Mitomycin-Mediated Cytotoxicity. Cell Res..

[18] Metalli D., Lovat F., Tripodi F., Genua M., Xu S.-Q., Spinelli M., Alberghina L., Vanoni M., Baffa R., Gomella L.G. (2010). The Insulin-Like Growth Factor Receptor I Promotes Motility and Invasion of Bladder Cancer Cells through Akt- and Mitogen-Activated Protein Kinase-Dependent Activation of Paxillin. Am. J. Pathol..

[19] Halstead A.M., Kapadia C.D., Bolzenius J., Chu C.E., Schriefer A., Wartman L.D., Bowman G.R., Arora V.K. (2017). Bladder-Cancer-Associated Mutations in RXRA Activate Peroxisome Proliferator-Activated Receptors to Drive Urothelial Proliferation. eLife.

[20] Rochel N., Krucker C., Coutos-Thévenot L., Osz J., Zhang R., Guyon E., Zita W., Vanthong S., Hernandez O.A., Bourguet M. (2019). Recurrent Activating Mutations of PPARγ Associated with Luminal Bladder Tumors. Nat. Commun..

[21] Chan M.W.Y., Chan L.W., Tang N.L.S., Tong J.H.M., Lo K.W., Lee T.L., Cheung H.Y., Wong W.S., Chan P.S.F., Lai F.M.M. (2002). Hypermethylation of Multiple Genes in Tumor Tissues and Voided Urine in Urinary Bladder Cancer Patients. Clin. Cancer Res. Off. J. Am. Assoc. Cancer Res..

[22] Waliszewski P., Waliszewska M.K., Hemstreet G.P., Hurst R.E. (1997). Expression of Sex Steroid Receptor Genes and Comodulation with Retinoid Signaling in Normal Human Uroepithelial Cells and Bladder Cancer Cell Lines. Urol. Oncol. Semin. Orig. Investig..

[23] Lin G., Zhu S., Wu Y., Song C., Wang W., Zhang Y., Chen Y.-L., He Z. (2017). ω-3 Free Fatty Acids and All-Trans Retinoic Acid Synergistically Induce Growth Inhibition of Three Subtypes of Breast Cancer Cell Lines. Sci. Rep..

