# Supplementary material for: Docosahexaenoic Acid Reverted the All-trans Retinoic Acid-Induced Cellular Proliferation of T24 Bladder Cancer Cell Line
Source: J Clin Med. 2020 Aug 3;9(8):2494. doi: 10.3390/jcm9082494 (PMC7465316; doi:10.3390/jcm9082494)
Supplement: Supplementary file 1 [file jcm-09-02494-s001.pdf]

Supplementary Materials:

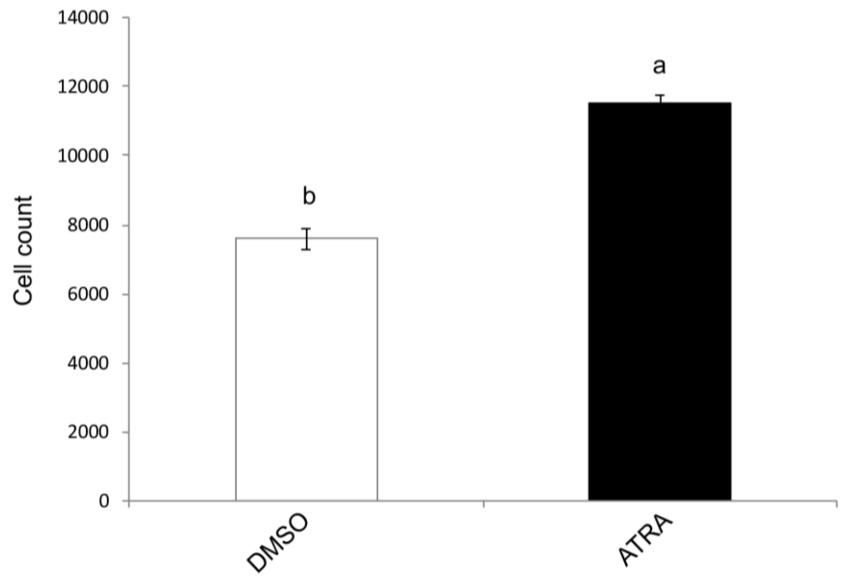

Figure S1. Absolute cell count made through flow-cytometry of T24 cells DMSO- and 10µM ATRA-treated at 72h. Means ± SD (n = 3). Means with different letters are significantly different ($p < 0.05$).

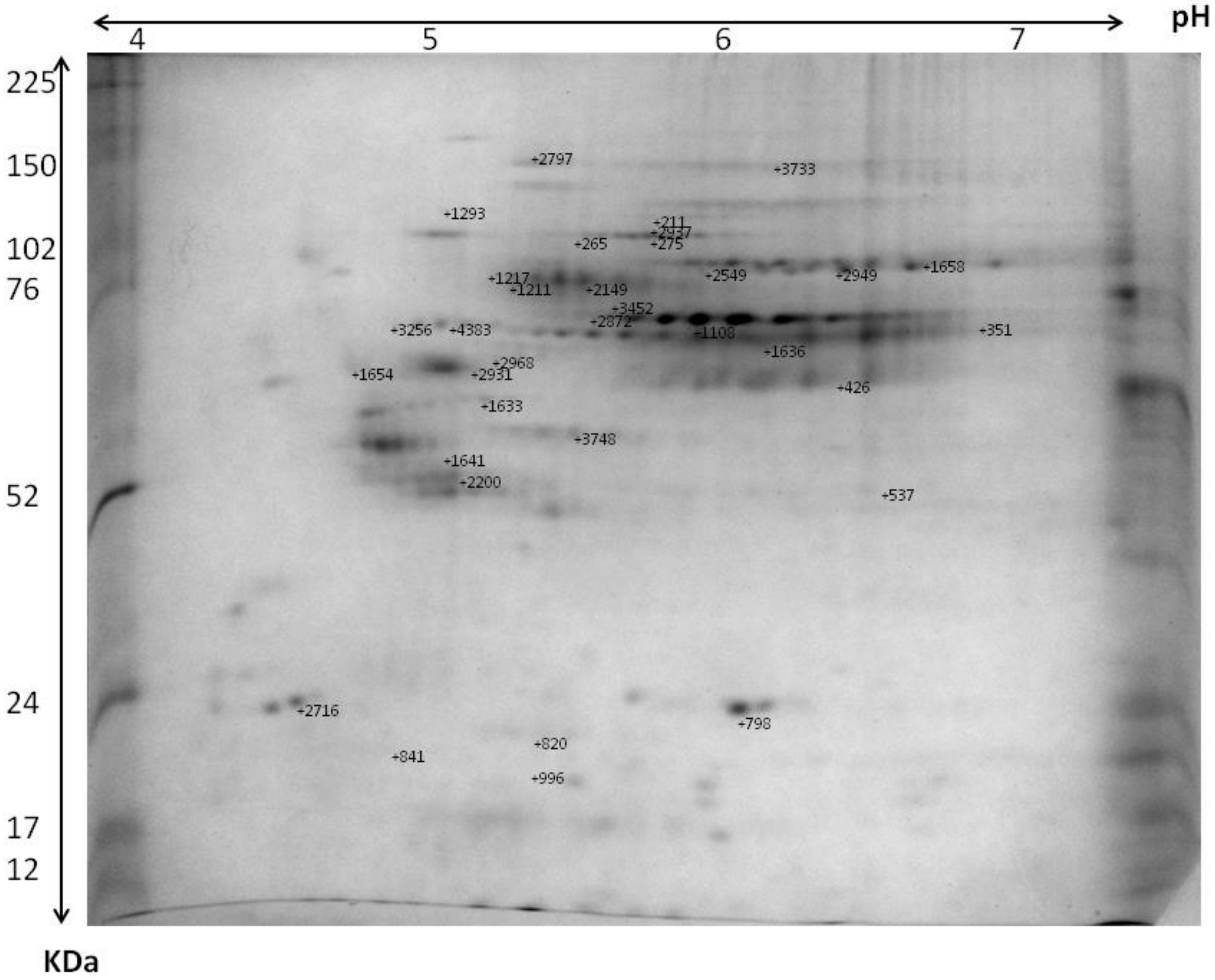

Figure S2. 2-DE Proteomic profiling of T24 cell line after 10µM ATRA treatment. The numbers indicate the protein spots as detected by using Progenesis SameSpots software, identified by mass spectrometry and detailed in Table S1.

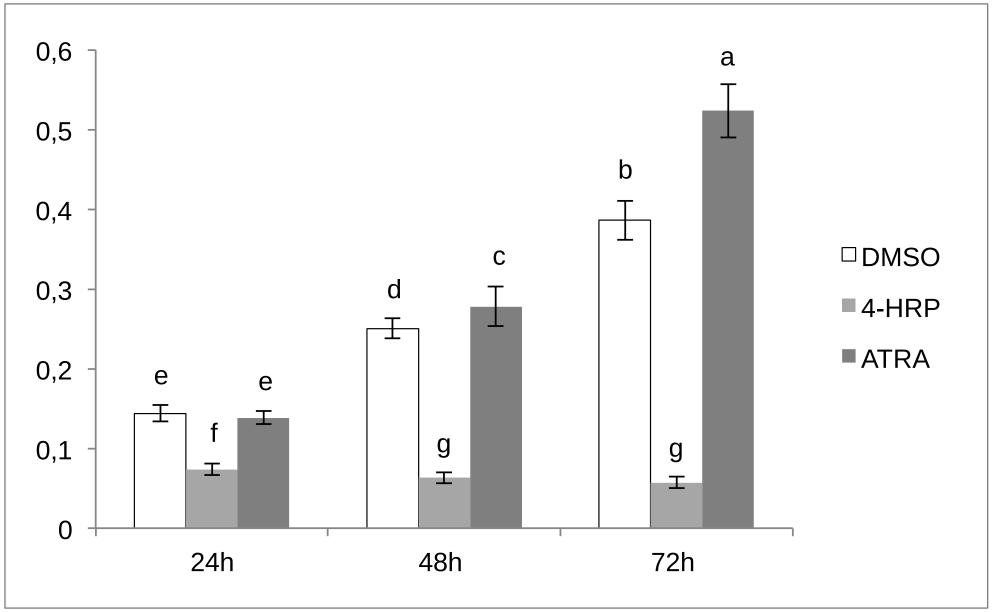

Figure S3. T24 are resistant to ATRA treatment but not to 4-HRP, synthetic retinoid. MTT assays express as OD (optical density) values in T24 cells treated with 10 μM of ATRA or respective concentration of vehicle (DMSO) (for 24 h, 48 h, 72 h), and with 10 μM of 4-HRP, synthetic retinoid. Means ± SD (n = 12). Means with different letters are significantly different ($p < 0.05$).

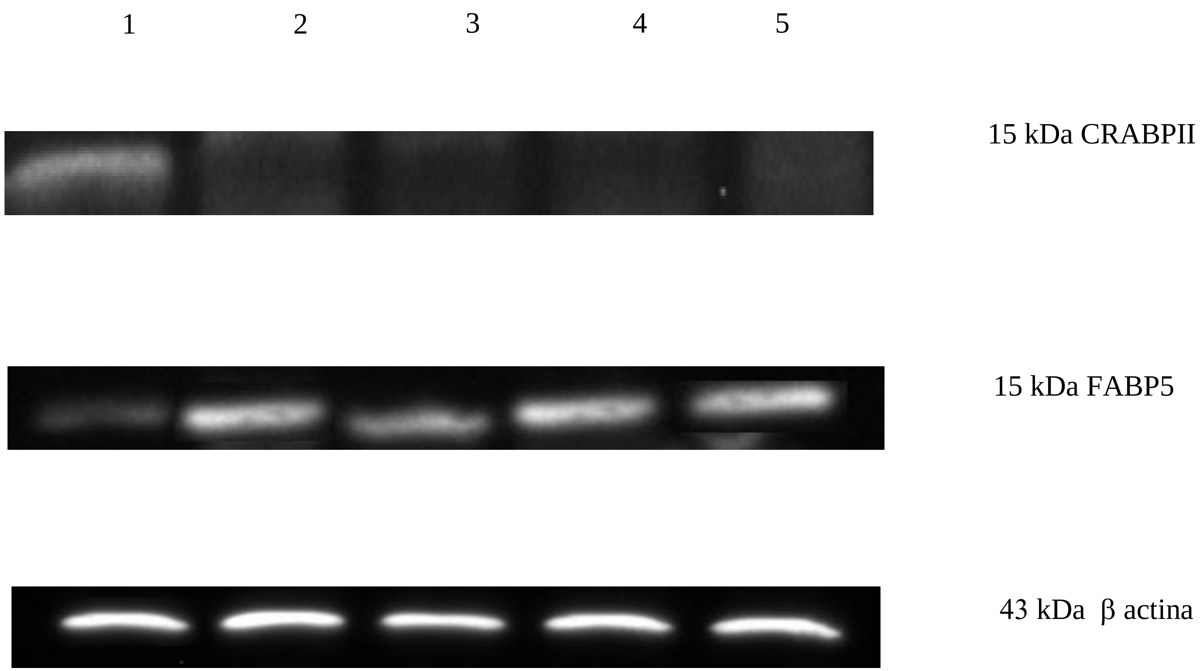

Figure S4. CRABP-II and FABP5 proteins' expression in total healthy bladder lysate (1), untreated T24 cells (2) 10 μM ATRA-treated T24 cells for 24 h (3), 48 h (4), and 72 h (5). β-actin was the control. Representative image out of three.

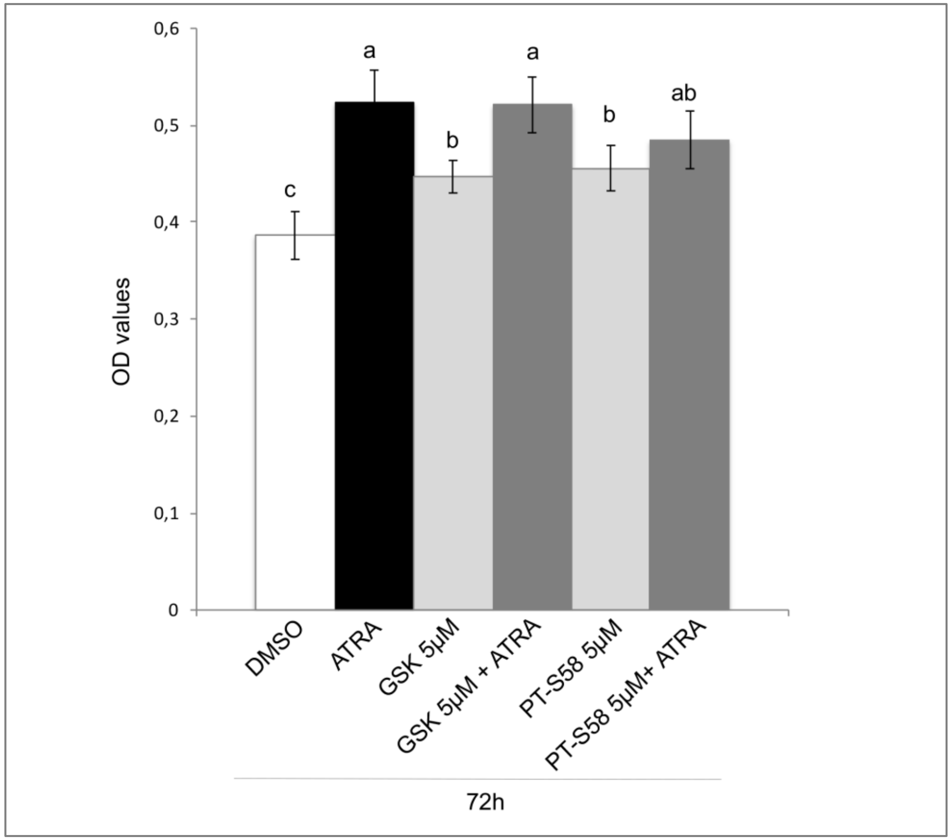

Figure S5. T24 are resistant to PPARβ/δ antagonists, GSK0660 and PTS-58, alone or in combination to ATRA. MTT assays express as OD (optical density) values in T24 cells treated with 10 µM of ATRA or DMSO (for 72 h), and with 5 µM of GSK or PT-S58 alone or in combination with 10 µM of ATRA. Means ± SD (n = 12). Means with different letters are significantly different ($p < 0.05$).

Table S1. List of differentially abundant proteins identified by LC-MS/MS T24 cells DMSO and 10 μM ATRA treated for three days. [a] Spot numbers refer to Supplementary Figure S2. [b] Values between parentheses indicate statistically significant peptides ($p < 0.05$). [c] Fold of protein variation refers to treated *vs.* control. D: down-regulated; U: up-regulated.

Spot No.[a]	Accession No.	STRING Accession No.	Protein name	Mascot score	Match[b]	Unique sequences[b]	Seq. coverage	Theoretical/Exp. Mr (kDa)	Theoretical/Exp. pI	Fold change[c]	One-way ANOVA (p-value)	Trend
1503	HS90B	HSP90AB1	Heat shock protein HSP 90-beta OS=Homo sapiens OX=9606 GN=HSP90AB1 PE=1 SV=4	344	28◎13◎	20(10)	30%	83554	4.97	2.4	0.003	D
3733	HSP7C	HSPA8	Heat shock protein cognate 71 kDa protein OS=Homo sapiens OX=9606 GN=HSPA8 PE=1 SV=1	709	77(27)	28(19)	40%	71082	5.37	1.8	0.002	D
275	CH60	HSPD1	60 kDa heat shock protein, mitochondrial OS=Homo sapiens OX=9606 GN=HSPD1 PE=1 SV=2	3668	306(169)	39(33)	65%	61187	5.70	1.5	0.031	D
1641	TPD54	TPD52L2	Tumor protein D54 OS=Homo sapiens OX=9606 GN=TPD52L2 PE=1 SV=2	54	4(2)	4(2)	28%	22281	5.26	3.8	0.034	D
2872	NUDC	NUDC	Nuclear migration protein nudC OS=Homo sapiens OX=9606 GN=NUD PE=1 SV=1	499	42(18)	22(15)	45%	38276	5.27	2.2	0.038	D
996	COTL1	COTL1	Coactosin-like protein OS=Homo sapiens OX=9606 GN=COTL1 PE=1 SV=3	121	17(6)	10(5)	46%	16049	5.54	2.2	0.037	D
2931	NPM	NPM1	Nucleophosmin OS=Homo sapiens OX=9606 GN=NPM1 PE=1 SV=2	535	46(17)	13(9)	39%	32726	4.64	1.8	0.009	D
3748	ANXA5	ANXA5	Annexin A5 OS=Homo sapiens OX=9606 GN=ANXA5 PE=1 SV=2	982	117(43)	23(16)	72%	35971	4.94	1.5	0.041	D
1636	POTEE	POTEE	POTE ankyrin domain family member E OS=Homo sapiens OX=9606 GN=POTEE PE=2 SV=3	1083	75(40)	11(6)	11%	122882	5.83	2.5	0.013	D
4417	ACTG	ACTG1	Actin, cytoplasmic 2 OS=Homo sapiens OX=9606 GN=ACTG1 PE=1 SV=1	1146	169(62)	26(20)	72%	42108	5.31	2.3	0.014	D
351	SEPT2	SEPT2	Septin-2 OS=Homo sapiens OX=9606 GN=SEPT2 PE=1 SV=1	169	10(4)	9(4)	26%	41689	6.15	1.8	0.014	D
265	LMNB1	LMNB1	Lamin-B1 OS=Homo sapiens OX=9606 GN=LMNB1 PE=1 SV=2	209	20(6)	15(6)	27%	66653	5.11	1.5	0.02	D
2549	TBA1B	TUBA1B	Tubulin alpha-1B chain OS=Homo sapiens OX=9606 GN=TUBA1B PE=1 SV=1	167	10(4)	6(4)	18%	50804	4.94	2.2	0.032	D
2968	HNRPC	HNRNPC	Heterogeneous nuclear ribonucleoproteins C1/C2 OS=Homo sapiens OX=9606 GN=HNRNPC PE=1 SV=4	55	4(1)	3(1)	10%	33707	4.95	1.5	0,025	D
11	PLIN3	PLIN3	Perilipin-3 OS=Homo sapiens OX=9606	196	7(3)	6(3)	18%	47217	5.30	1.7	0.027	D

08			GN=PLIN3 PE=1 SV=3									
2149	ATPB	ATP5B	ATP synthase subunit beta, mitochondrial OS=Homo sapiens OX=9606 GN=ATP5F1B PE=1 SV=3	2051	206(93)	22(17)	53%	56525	5.26	1.8	0.03	D
3669	BIP	HSPA5	Endoplasmic reticulum chaperone BiP OS=Homo sapiens OX=9606 GN=HSPA5 PE=1 SV=2	1312	109(47)	37(28)	54%	72402	5.07	2.0	0.046	D
841	MYL6	MYL6	Myosin light polypeptide 6 OS=Homo sapiens OX=9606 GN=MYL6 PE=1 SV=2	770	90(31)	10(8)	68%	17090	4.56	1.9	0.029	D
2200	1433B	YWHAB	14-3-3 protein beta/alpha OS=Homo sapiens OX=9606 GN=YWHAB PE=1 S	158	11(6)	7(5)	26%	28179	4.76	1.8	0.032	D
1217	TBB5	TUBB	Tubulin beta chain OS=Homo sapiens OX=9606 GN=TUBB PE=1 SV=2	285	26(12)	16(10)	38%	50095	4.78	2.1	0.036	D
211	CALD1	CALD1	Caldesmon OS=Homo sapiens OX=9606 GN=CALD1 PE=1 SV=3	51	7(2)	7(2)	10%	93232	5.62	4.0	0.038	D
3452	NSF1C	NSFL1C	NSFL1 cofactor p47 OS=Homo sapiens OX=9606 GN=NSFL1C PE=1 SV=2	234	10(5)	8(5)	24%	40548	4.99	3.0	0.043	D
1211	CAVN1	PTRF	Caveolae-associated protein 1 OS=Homo sapiens OX=9606 GN=CAVIN1 PE=1 SV=1	224	24(7)	9(6)	20%	43450	5.51	2.5	0.045	D
2797	1433E	YWHAE	14-3-3 protein epsilon OS=Homo sapiens OX=9606 GN=YWHAE PE=1 S	199	16(6)	9(5)	40%	29326	4.63	6.7	0.046	D
2973	LMNB2	LMNB2	Lamin-B2 OS=Homo sapiens OX=9606 GN=LMNB2 PE=1 SV=4	313	36(11)	25(9)	37%	70020	5.50	2.0	0.05	D
1293	PDIA1	P4HB	Protein disulfide-isomerase OS= Homo sapiens OX= 9606 GN=P4HB PE=1 SV=3	97	7(2)	6(2)	11%	57480	4.76	1.6	0.002	U
2608	TPM1	TPM1	Tropomyosin alpha-1 chain OS=Homo sapiens OX= 9606 GN=TPM1 PE= 1 SV=2	668	92(32)	24(14)	56%	32746	4.69	2.6	0.048	U
426	ACTB	ACTB	Actin, cytoplasmic 1 OS=Homo sapiens OX=9606 GN=ACTB PE=1 SV=1	1017	148(57)	23(16)	67%	42052	5.29	2.2	0.017	U
798	STMN1	STMN1	Stathmin OS=Homo sapiens OX=9606 GN=STMN1 PE=1 SV=3	310	47(17)	16(8)	70%	17292	5.76	1.7	0.009	U
2949	PDIA3	PDIA3	Protein disulfide-isomerase A3 OS=Homo sapiens OX=9606 GN=PDIA3 PE=1	305	22(10)	16(10)	31%	57146	5.98	2.5	0.009	U
820	IF5A1	EIF5A	Eukaryotic translation initiation factor 5A-1 OS=Homo sapiens OX=9606 GN=EIF5A PE=1 SV=2	56	7(2)	5(1)	20%	17049	5.08	1.5	0.010	U
1658	TCPB	CCT2	T-complex protein 1 subunit beta OS=Homo sapiens OX=9606 GN=CCT2 PE=1 SV=4	595	42(19)	26(16)	58%	57794	6.01	1.5	0.011	U

53 7	PHB	PHB	Prohibitin OS=Homo sapiens OX=9606 GN=PHB PE=1 SV=1	334	30(15)	11(8)	36%	29843	5.57	2.1	0.011	U
16 54	TPM2	TPM2	Tropomyosin beta chain OS=Homo sapiens OX=9606 GN=TPM2 PE=1 SV=1	657	48(28)	23(16)	51%	32945	4.66	2.0	0.012	U
32 56	ALBU	ALB	Serum albumin OS=Homo sapiens OX=9606 GN=ALB PE=1 SV=2	79	11(3)	7(3)	8%	71317	5.92	1.5	0.023	U
43 83	RSSA	RPSA	40S ribosomal protein SA OS=Homo sapiens OX=9606 GN=RPSA PE=1 SV=4	449	51(28)	9(6)	32%	32947	4.79	1.5	0.034	U
16 33	EF1D	EEF1D	Elongation factor 1-delta OS=Homo sapiens OX=9606 GN=EEF1D PE=1 SV=5	191	15(6)	10(4)	43%	31217	4.90	1.6	0.035	U
27 16	RLA2	RPLP2	60S acidic ribosomal protein P2 OS=Homo sapiens OX=9606 GN=RPLP2 PE=1 SV=1	624	43(17)	4(3)	53%	11658	4.42	2.0	0,043	U
